# Antimicrobial and inflammatory properties of South African clinical *Lactobacillus* isolates and vaginal probiotics

**DOI:** 10.1038/s41598-018-38253-4

**Published:** 2019-02-13

**Authors:** Emily Chetwin, Monalisa T. Manhanzva, Andrea G. Abrahams, Remy Froissart, Hoyam Gamieldien, Heather Jaspan, Shameem Z. Jaumdally, Shaun L. Barnabas, Smritee Dabee, Anna-Ursula Happel, Desiree Bowers, Lester Davids, Jo-Ann S. Passmore, Lindi Masson

**Affiliations:** 1 Institute of Infectious Disease and Molecular Medicine (IDM), University of Cape Town, Cape Town, South Africa; 2 UMR 5290 MIVEGEC, French National Centre for Scientific Research (CNRS), Montpellier, France; 3 Seattle Children’s Research Institute, University of Washington, Seattle, Washington USA; 4 Department of Human Biology, University of Cape Town, Cape Town, South Africa; 5 Centre for the AIDS Programme of Research in South Africa (CAPRISA) Centre of Excellence in HIV Prevention, Durban, South Africa; 6 National Health Laboratory Service, Cape Town, South Africa

## Abstract

Bacterial vaginosis (BV) causes genital inflammation and increased HIV acquisition risk. The standard-of-care for BV, antibiotic therapy, is associated with high recurrence rates. Probiotics may improve treatment outcomes, although substantial heterogeneity in efficacy has been observed during clinical trials. To evaluate the potential to improve existing probiotics, we compared the inflammatory and antimicrobial (adhesion, H_2_O_2_, D-lactate and L-lactate production) characteristics of 23 vaginal *Lactobacillus* isolates from South African women, commercial vaginal probiotics (*L. casei rhamnosus*, *L. acidophilus*) and 4 reference strains. All lactobacilli induced inflammatory cytokine production by genital epithelial cells and produced D-lactate. Of six isolates assessed, five suppressed inflammatory responses to *Gardnerella vaginalis*. Although the *L. acidophilus* probiotic was the most adherent, many clinical isolates produced greater amounts of H_2_O_2_, D-lactate and L-lactate than the probiotics. The most L-lactate and H_2_O_2_ were produced by *L. jensenii* (adjusted p = 0.0091) and *L. mucosae* (adjusted p = 0.0308) species, respectively. According to the characteristics evaluated, the top 10 isolates included 4 *L. jensenii*, 2 *L. crispatus*, 1 *L. mucosae*, 1 *L. vaginalis* and the *L. acidophilus* probiotic. There is potential to develop an improved vaginal probiotic using clinical *Lactobacillus* isolates. Inflammatory profiles are critical to evaluate as some isolates induced substantial cytokine production.

## Introduction

Bacterial vaginosis (BV) is a highly prevalent dysbiosis of the vaginal microbiota that is characterized by a shift from predominantly *Lactobacillus* species to a diverse population including pathogenic bacteria, such as *Gardnerella vaginalis* and *Prevotella spp*^1^. BV has been found to increase the risk of HIV acquisition in women, mother-to-child transmission and transmission from women with BV to their male partners, as well as reduce the efficacy of some forms of antiretroviral pre-exposure prophylaxis^2–6^. Additionally, BV is associated with increased susceptibility to other sexually transmitted infections (STIs) and severe reproductive complications in women^7,8^. Although the underlying mechanisms are not fully understood, these outcomes may be partly mediated by increases in vaginal pro-inflammatory cytokine concentrations and immune cell activation in the female genital tracts (FGTs) of women with BV^9^. The relationship between BV and HIV is particularly concerning in South Africa, where approximately 18% of the adult population aged 15–49 years is HIV-infected^10^ and BV prevalence rates are reported to exceed 50% in some regions^11^.

*Lactobacillus* species, which dominate in a healthy FGT, are thought to protect against BV, HIV and other STIs by a number of mechanisms. Lactic acid produced by lactobacilli hinders the growth of potential pathogens and inactivates HIV^12,13^, partly by maintaining the physiological pH of the vagina below 4.5^14^. Lactic acid exists as both D- and L-isomers; while L-lactic acid has been found to inactivate HIV more effectively than D-lactic acid^15^, D-lactic acid is thought to play a more important role in inhibiting bacterial pathogens, including *Chlamydia trachomatis*^16,17^. Many lactobacilli also produce hydrogen peroxide (H_2_O_2_), which has a virucidal effect on HIV by inhibiting viral adhesion and replication^18^. However, the role of H_2_O_2_ in protection against BV-associated bacteria is controversial, as some studies have reported that physiological concentrations are not microbicidal and that, at microbicidal concentrations, H_2_O_2_ inhibits lactobacilli more effectively than pathogenic bacteria^19^. Competitive exclusion is another important protective mechanism utilized by lactobacilli, whereby adherent lactobacilli prevent the adhesion of pathogens to the vaginal epithelium and thus colonization^20^. Genital inflammation caused by BV and STIs increases risk of HIV acquisition^21^. Suppression of inflammatory responses by lactobacilli and lactic acid is another proposed mechanism for reduced susceptibility to HIV in women with *Lactobacillus*-dominant microbiota^22^.

As the standard-of-care for BV, antibiotic treatment, is associated with high recurrence rates^14,23^, there is an urgent need to develop better treatment strategies, particularly in regions of high HIV prevalence. Several randomized clinical trials have evaluated *Lactobacillus*-containing probiotics for BV treatment, alone or as adjunctive therapy with antibiotics^24,25^. Although some trials have demonstrated improved BV outcomes with probiotics, the results have been heterogeneous, with some studies finding no benefit^24,25^. One of the limitations of many of these probiotic formulations is that they include bacterial species that are not adapted for survival in the FGT and are not usually found in women with healthy vaginal microbiota^26^. It is also possible that probiotics containing *Lactobacillus* isolates from one population may have reduced efficacy when used in another population, as major geographical and ethnic differences have been observed in the vaginal microbiota and host factors that may influence bacterial colonization^27,28^. Therefore, using vagina-specific *Lactobacillus* species with effective antimicrobial properties that have been isolated from within the population of intended use may improve BV treatment outcomes. The aims of this study were to compare the antimicrobial and inflammatory characteristics of existing vaginal probiotics on the South African market to those of clinical *Lactobacillus* isolates from the FGTs of South African women.

## Results

### Study population and *Lactobacillus* isolates

Clinical *Lactobacillus* strains (n = 23) were isolated from cervicovaginal samples from nine women residing in Cape Town, South Africa (Table 1)^29^. Of these, six women had no STIs or BV, two had BV and one was infected with *Chlamydia trachomatis*. All of the women were PCR negative for other STIs including: *Neisseria gonorrhoeae, Trichomonas vaginalis, Mycoplasma genitalium*, herpes simplex virus (HSV)-1, HSV-2 and *Treponema pallidum*. The 23 clinical *Lactobacillus* isolates included seven *L. crispatus* (LC1–7), one *L. gasseri* (LG1), five *L. jensenii* (LJ1–5), four *L. mucosae* (LM1–4) and six *L. vaginalis* (LV1–6). Two commercial vaginal probiotics were found on the South African market and lactobacilli isolated from these probiotics were evaluated. One probiotic contained *L. acidophilus* in vaginal tablet and oral capsule formulations, and the other contained *L. casei rhamnosus* Lcr35 in a vaginal capsule. Four American Type Culture Collection (ATCC) reference strains [33197 (*L. crispatus)*, 33820 (*L. crispatus)*, 9857 (*L. gasseri)*, 25258 (*L. jensenii*)] were also included.

**Table 1:** Demographic and clinical characteristics of study participants.

Demographic characteristics	n (%)
Black race	9 (100)
Median age in years (range)	18 (17–21)
**Clinical and laboratory findings**	**n (%)**
No STI or bacterial vaginosis	6 (66.7)
*Chlamydia trachomatis* (PCR positive)	1 (11.1)
*Neisseria gonorrhoeae* (PCR positive)	0 (0)
*Trichomonas vaginalis* (PCR positive)	0 (0)
*Mycoplasma genitalium* (PCR positive)	0 (0)
HSV-2 IgG	0 (0)
HSV (PCR positive)	0 (0)
*Treponema pallidum* (RPR > 1:4, TPHA positive)	0 (0)
Bacterial vaginosis (Nugent score ≥ 7)	2 (22.2)
Intermediate flora (Nugent score 4–6)	0 (0)
Yeast cells	1 (11.1)
Any STI or bacterial vaginosis	3 (33.3)
Abnormal vaginal discharge	1 (11.1)
Median vaginal swab pH (range)	4.7 (4.1–5.3)
Median soft-cup pH (range)	4.3 (3.6–5.6)
**Contraception**	
*Petogen	2 (22.2)
*Nur-Isterate	7 (77.8)

STI, sexually transmitted infection; PCR, polymerase chain reaction; HSV-2, herpes simplex virus type 2; RPR, rapid plasma reagin; TPHA, *Treponema pallidum* hemagglutination. *Progesterone-based injectables.

**Figure 1:**
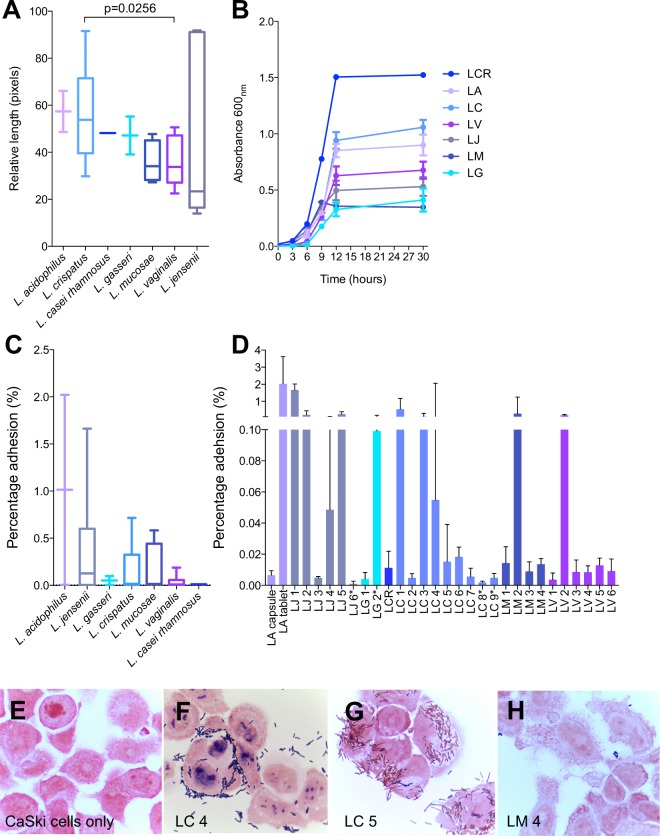
Size, growth rates and adhesion of *Lactobacillus* isolates. (**A**) Bacterial length was evaluated using microscopy and lengths of different isolates grouped by species are shown, with species ordered from largest to smallest. Lines indicate medians of species (5 measurements per isolate), bars indicate the interquartile ranges and error bars indicate the ranges. (**B**) Growth rates were evaluated by inoculating MRS broth with 4.18 × 10^6^ colony forming units (CFU) of each isolate, incubating anaerobically at 37 °C, and measuring the optical densities (600 nm) of cultures at various time-points. Growth rates by species are shown with symbols indicating means and error bars indicating the standard errors of the means of different isolates of the same species. (**C–H**) *Lactobacillus* adhesion to CaSki (ectocervical epithelial) cells was assessed by adding optical density (OD)-adjusted bacteria (OD_600_ 0.1 ± 0.01) to cell monolayers, incubating for 2 h and washing to remove unbound bacteria. (**C**) Adhesion was evaluated in three separate experiments for each isolate. Following addition of bacteria and a 2 h incubation period, cells were lifted and plated on MRS agar and colony-forming units were counted. Adhesion is expressed as the percentage of the number of bacteria added to the monolayers that remained adherent. Different isolates of the same species were grouped together and species are ordered from the most to least adhesive. Lines indicate medians, bars indicate the interquartile ranges and error bars indicate the ranges. (**D**) The level of adhesion of each isolate is shown separately, with bars indicating medians and error bars indicating the ranges of adhesion evaluated in three separate experiments. (**E–H**) Adhesion was assessed qualitatively by Gram staining and collecting images at 1000x magnification using a light microscope. Mann-Whitney test was used for comparisons between species and p-values < 0.05 after adjustment for multiple comparisons were considered statistically significant. LA: *Lactobacillus acidophilus* probiotic (n = 2); LC: *Lactobacillus crispatus* (n = 9); LCR: *Lactobacillus casei rhamnosus* probiotic (n = 1); LG: *Lactobacillus gasseri* (n = 2); LM: *Lactobacillus mucosae* (n = 4); LV: *Lactobacillus vaginalis* (n = 6); LJ: *Lactobacillus jensenii* (n = 6). *ATCC reference strains.

The sizes and growth rates (under anaerobic conditions) of the clinical and probiotic lactobacilli, both inter- and intra-species were varied (Fig. 1). The *L. acidophilus* probiotic isolate tended to be larger than the majority of the clinical isolates, with the exception of some of the *L. crispatus* and *L. jensenii* isolates (Fig. 1A). The probiotic isolate *L. casei rhamnosus* grew most rapidly, followed by *L. crispatus* isolates and the *L. acidophilus* probiotic (Fig. 1B), although these differences were not statistically significant.

### *Lactobacillus* adhesion to genital epithelial cells

*Lactobacillus* isolates were tested for their ability to adhere to CaSki (ectocervical epithelial) cells (Fig. 1C–H). Overall, the *L. acidophilus* probiotic isolate was the most adherent, although there was a large difference between the adhesion of the isolate obtained from the capsule (0.006%) versus the isolate obtained from the tablet (2.022%) (Fig. 1C,D). Although there were no statistically significant differences in adhesion between species (Fig. 1C), among the clinical isolates, *L. jensenii* 1 (LJ1) exhibited the greatest adhesion, with approximately 1.663% of bacteria adhering to the cells (Fig. 1D). Several species included a relatively large proportion of highly adherent strains, with 1/2 (50%) *L. acidophilus*, 4/6 (67%) *L. jensenii*, and 1/2 (50%) *L. gasseri* relatively highly adherent. It has previously been suggested that bacterial size and growth rates may influence adhesion ability^30^. However, neither bacterial size nor growth rates correlated with *Lactobacillus* adhesion in this study (Spearman rho = 0.2209, p = 0.2407 and rho = 0.2843, p = 0.1278, respectively; data not shown).

### Hydrogen peroxide production

**Figure 2:**
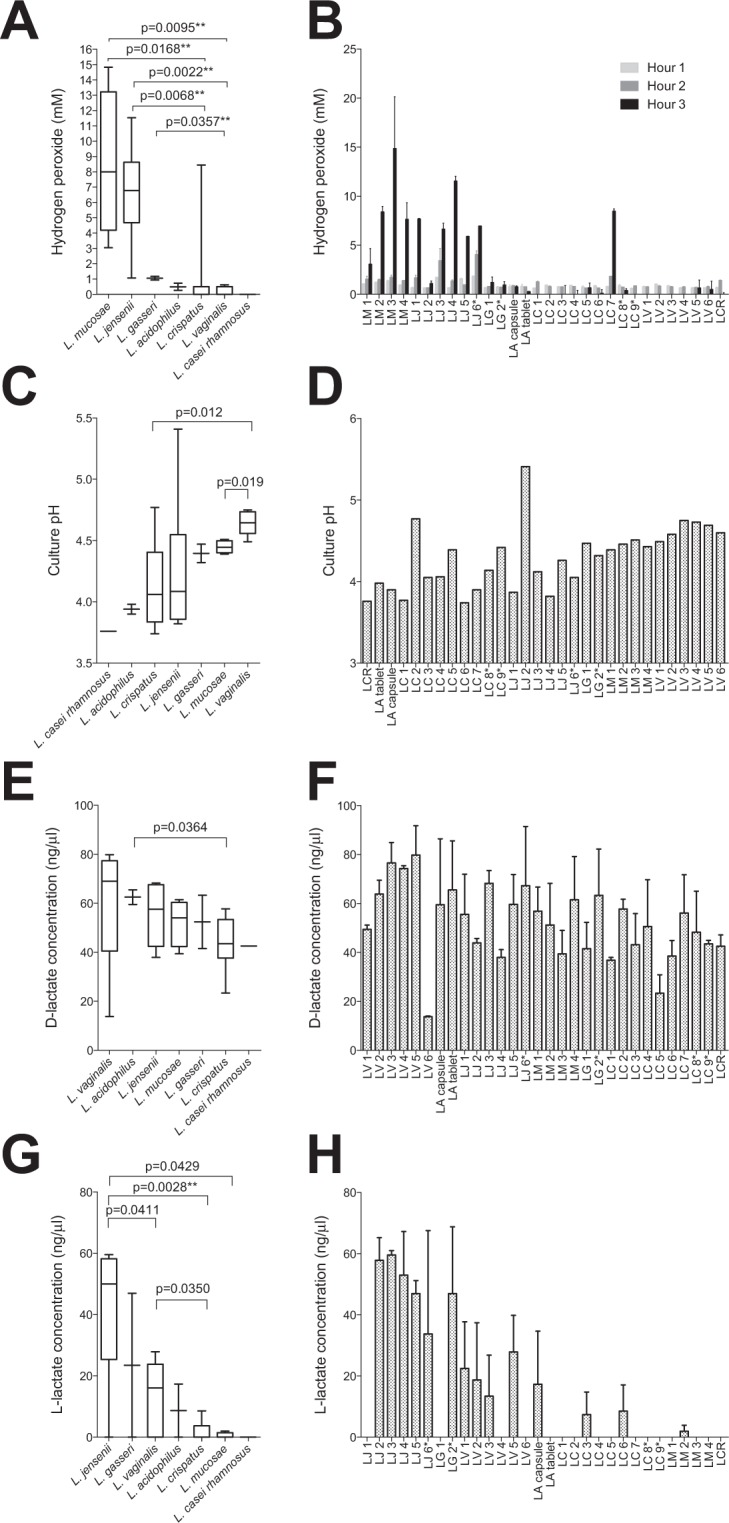
Antimicrobial characteristics of *Lactobacillus* isolates. (**A**,**C**,**E**,**G**) Different isolates of the same species were grouped together and the overall antimicrobial properties of different species are shown, with species ordered according to the amounts of hydrogen peroxide (H_2_O_2_), D- and L-lactate produced and culture pH. Lines indicate medians, bars indicate the interquartile ranges and error bars indicate the ranges. (**B**,**D**,**F**,**H**) Antimicrobial properties for individual isolates are also shown with bars indicating the medians and error bars indicating the ranges of technical replicates within each assay. (**A**,**B**) Hydrogen peroxide production after 3 h of aerobic culture was measured in duplicate within the same assay. (**C**,**D**) pH levels of bacterial cultures were measured using a pH meter following anaerobic culture at 37 °C for 24 hours. (**E**,**F**) D-lactate and (**G**,**H**) L-lactate concentrations after a 24 h incubation period at 37 °C under anaerobic conditions were measured in duplicate within the same assays using Lactate Colorimetric kits. Mann-Whitney test was used for comparisons between species and p-values < 0.05 after adjustment for multiple comparisons were considered statistically significant. **Associations that remained statistically significant after adjustment for multiple comparisons. LA: *Lactobacillus acidophilus* probiotic (n = 2); LC: *Lactobacillus crispatus* (n = 9); LCR: *Lactobacillus casei rhamnosus* probiotic (n = 1); LG: *Lactobacillus gasseri* (n = 2); LM: *Lactobacillus mucosae* (n = 4); LV: *Lactobacillus vaginalis* (n = 6); LJ: *Lactobacillus jensenii* (n = 6). *ATCC reference strains.

*L. mucosae* isolates produced the most H_2_O_2_ relative to the other strains collectively (p = 0.0044; adjusted p = 0.0308), after a 3 h incubation period under aerobic conditions, followed by *L. jensenii* isolates (p = 0.0061; adjusted p = 0.0427; data not shown). The other species evaluated produced comparably lower amounts of H_2_O_2_ (Fig. 2A). Only one *L. crispatus* isolate, none of the *L. vaginalis* isolates and none of the probiotic *Lactobacillus* isolates produced significant amounts of H_2_O_2_ (Fig. 2B). The rates of H_2_O_2_ production were similar for all producing isolates, with production being evident within 2 h (Fig. 2B). The level of H_2_O_2_ production was not associated with the growth rates of the lactobacilli (rho = −0.1136, p = 0.5501; data not shown).

### D- and L-lactate production and culture acidification

When the lactobacilli were cultured in MRS, it was found that the probiotics lowered the culture pH levels most, followed by the *L. crispatus* isolates (Fig. 2C,D). In contrast*, L. vaginalis* and *L. mucosae* culture pH levels were the highest. Most *Lactobacillus* isolates produced large amounts of D-lactate, with no significant differences between species after multiple comparisons adjustment (Fig. 2E,F). L-lactate production was however highly varied between isolates (Fig. 2G,H), with *L. jensenii* isolates producing significantly greater amounts of L-lactate than the other isolates collectively (p = 0.0013, adjusted p = 0.0091; data not shown), followed by *L. gasseri* and *L. vaginalis* (Fig. 2G). While all the probiotic isolates produced large amounts of D-lactate, only the probiotic capsule *L. acidophilus* isolate produced detectable levels of L-lactate (Fig. 2F,H).

The concentration of total lactic acid, calculated using the Henderson-Hasselbalch equation^31^, correlated with culture pH (rho = 0.8626, p < 0.0001; data not shown). Additionally, there was a trend towards a significant correlation between D-lactate and L-lactate production (Spearman rho = 0.3272, p = 0.0776; data not shown). Although there was a trend towards an inverse correlation between culture pH and bacterial growth rates (rho = −0.3079, p = 0.0978), neither D-lactate (rho = 0.07787, p = 0.6825) nor L-lactate (rho = 0.2520, p = 0.1791) correlated with growth rates (data not shown).

### Inflammatory cytokine responses

**Figure 3:**
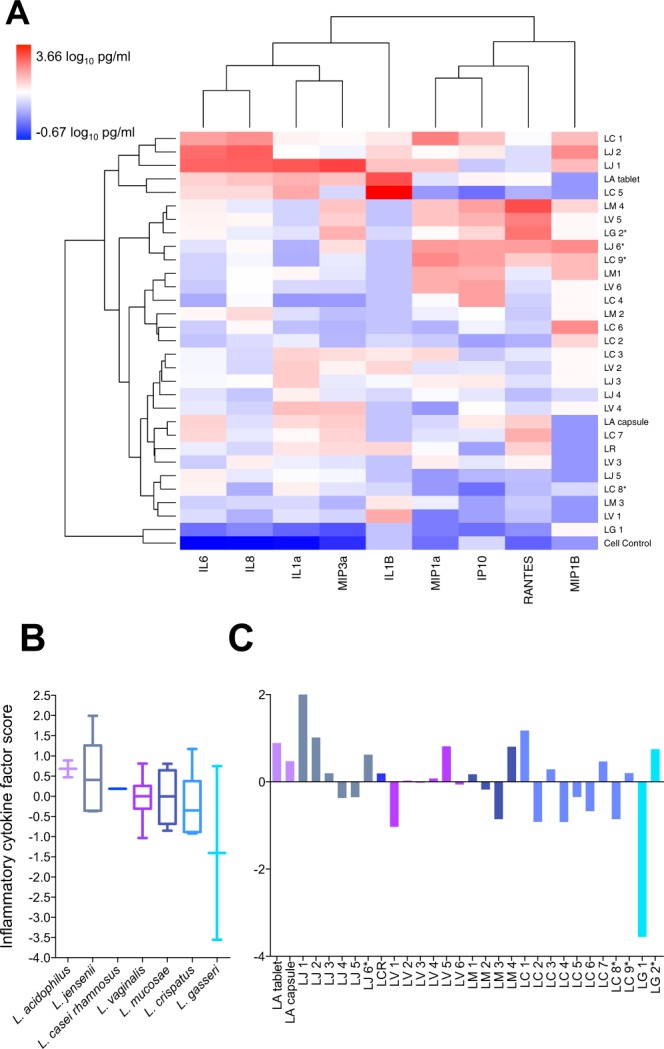
Inflammatory cytokine production by CaSki cells in response to *Lactobacillus* isolates. Cytokine production by CaSki cells in response to lactobacilli after a 24 h incubation period at 37 °C under 5% CO_2_ was measured using Luminex. (**A**) Unsupervised hierarchical clustering was used to group lactobacilli isolates according to inflammatory responses induced. Inflammatory cytokine concentrations are shown as a heat map, with blue, through white, to red indicating low-high cytokine concentrations, respectively. Data was log_10_-transformed and scaled in R. Two clustering dendrograms are shown in the figure. The dendrogram above the heat map illustrates degrees of relatedness between different cytokines measured. The dendrogram on the left hand side of the heat map indicates relationships between the expression profiles of the analysed cytokines in response to different clinical *Lactobacillus* isolates. (**B**) Different isolates of the same species were grouped together and overall inflammatory responses to each species were determined by grouping all 9 inflammatory cytokines measured onto 1 factor and generating factor scores for the isolates. Species are ordered from most to least inflammatory; lines indicate medians, bars indicate interquartile ranges and error bars indicate ranges. Mann-Whitney test was used for comparisons between species; no significant differences in inflammatory responses were observed between species. (**C**) Overall inflammatory responses to each isolate. LA: *Lactobacillus acidophilus* probiotic (n = 2); LC: *Lactobacillus crispatus* (n = 9); LCR: *Lactobacillus casei rhamnosus* probiotic (n = 1); LG: *Lactobacillus gasseri* (n = 2); LM: *Lactobacillus mucosae* (n = 4); LV: *Lactobacillus vaginalis* (n = 6); LJ: *Lactobacillus jensenii* (n = 6). *ATCC reference strains.

Inflammatory cytokines produced by CaSki cells in response to 4.18 × 10^6^ colony forming units (CFU) of each *Lactobacillus* isolate were assessed using Luminex. While several cytokines [including interleukin (IL)-1α, IL-1β, IL-6, IL-8, IFN-γ-inducible protein (IP)-10, macrophage inflammatory protein (MIP)-1α, MIP-1β, MIP-3α, regulated on activation, normal T cell expressed and secreted (RANTES)] were induced, others [including tumor necrosis factor (TNF)-α, interferon (IFN)-γ, IL-17A and IL-10] were not produced (below the minimum levels of detection for all samples; not shown). The relative cytokine production elicited by each of the isolates is shown as a heatmap (Fig. 3A). While most lactobacilli induced very little cytokine production by CaSki cells, some of the isolates were substantially more inflammatory than others, including two *L. crispatus* isolates, two *L. jensenii* isolates and the *L. acidophilus* vaginal tablet isolate which clustered separately from the other isolates assessed (Fig. 3A). Factor analysis was used to group all of the inflammatory cytokines together onto one factor and generate overall inflammatory scores for each isolate (Fig. 3B,C). *L. acidophilus* was most inflammatory, while *L. gasseri* isolates were the least inflammatory according to inflammatory factor scores (Fig. 3B), although these differences were not statistically significant. Of the individual isolates assessed, LJ1 was the most inflammatory, while LG1 was the least inflammatory (Fig. 3C).

**Figure 4:**
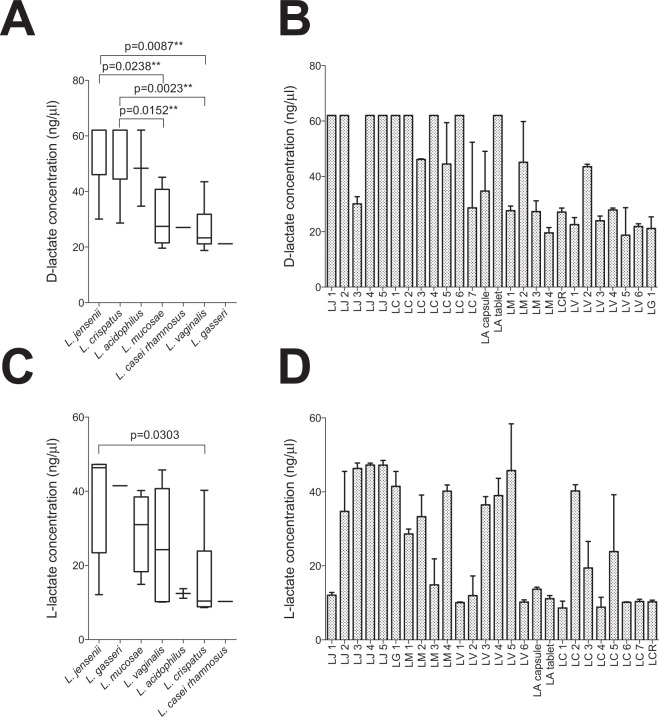
L-lactate and D-lactate production by lactobacilli after a 24 h incubation period with CaSki cells at 37 °C under 5% CO_2_. D-lactate and L-lactate concentrations after a 24 h incubation period at 37 °C under 5% CO_2_ in antibiotic-free cell culture medium were measured in duplicate within the same assays using Lactate Colorimetric kits. (**A**) D- and (**C**) L-lactate production by isolates grouped according to species are shown. Lines indicate medians, bars indicate the interquartile ranges and error bars indicate the ranges. (**B**,**D**) Lactate production by individual isolates is also shown with bars indicating the medians and error bars indicating the ranges of technical replicates within each assay. Mann-Whitney test was used for comparisons between species and p-values < 0.05 after adjustment for multiple comparisons were considered statistically significant. **Associations that remained statistically significant after adjustment for multiple comparisons. LA: *Lactobacillus acidophilus* probiotic (n = 2); LC: *Lactobacillus crispatus* (n = 7); LCR: *Lactobacillus casei rhamnosus* probiotic (n = 1); LG: *Lactobacillus gasseri* (n = 1); LM: *Lactobacillus mucosae* (n = 4); LV: *Lactobacillus vaginalis* (n = 6); LJ: *Lactobacillus jensenii* (n = 5).

To evaluate the impact of D- and L-lactate concentrations on inflammatory responses, these metabolites were measured in lactobacilli-CaSki co-cultures following a 24 hour incubation at 37 °C under aerobic conditions (Fig. 4). It was found that neither D-lactate nor L-lactate correlated significantly with inflammatory cytokine production by CaSki cells (data not shown).

To validate these findings using an alternative experimental approach, cytokine responses to isolates adjusted to optical densities at a wavelength of 600 nm (OD_600_) of 0.1 ± 0.01 were evaluated. It was found that the cytokine factor scores between assays (OD-adjusted bacteria versus 4.18 × 10^6^ CFU of bacteria used for stimulation) were significantly associated [β-coefficient = 0.396, 95% confidence interval (CI): 0.036–0.756, p = 0.032], particularly after adjustment for bacterial length (β-coefficient = 0.462, 95% CI: 0.088–0.835, p = 0.017), which influences the OD of cultures.

**Figure 5:**
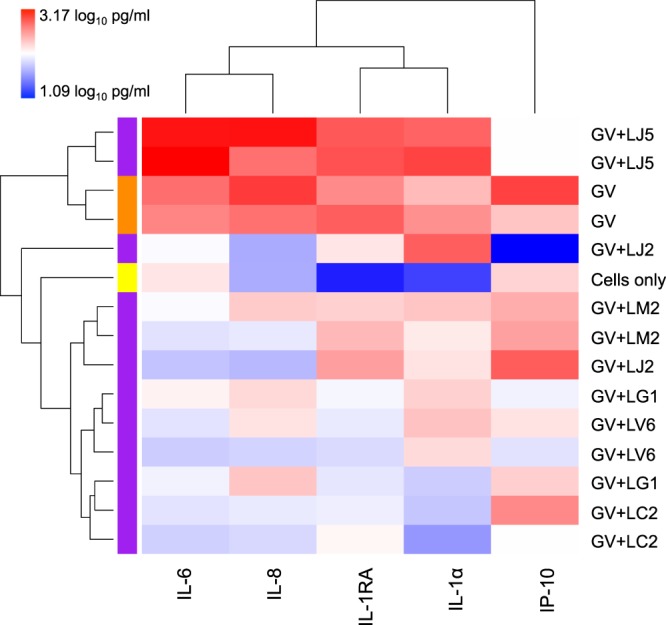
Lactobacilli-mediated changes in inflammatory cytokine production by CaSki cells in response to *Gardnerella vaginalis*. Cytokine production by CaSki cells in response to *G. vaginalis* alone and *G. vaginalis* following pre-incubation with lactobacilli. CaSki monolayers in 24 well plates were incubated with lactobacilli for 5 hours at 37 °C under 5% CO_2._ *G. vaginalis* was then added to the cultures and incubated as above for a further 20 hours. Cytokine concentrations were measured using Luminex. Unsupervised hierarchical clustering was used to group the co-cultures according to inflammatory responses induced. Inflammatory cytokine concentrations are shown as a heat map, with blue, through white, to red indicating low-high cytokine concentrations, respectively. Data was log_10_-transformed and scaled in R. Two clustering dendrograms are shown in the figure. The dendrogram above the heat map illustrates degrees of relatedness between different cytokines measured. The dendrogram on the left hand side of the heat map indicates relationships between the expression profiles of the analysed cytokines in response to different clinical *Lactobacillus* isolates. GV: *G*. *vaginalis*; LC: *L. crispatus*, LJ: *L. jensenii*; LM: *L. mucosae*; LG: *L. gasseri*; LV: *L. vaginalis*.

To evaluate the impact of lactobacilli on cytokine responses elicited by pathogenic, BV-associated bacteria, we pre-incubated CaSki cells with six *Lactobacillus* isolates [2 *L. jensenii* (LJ2 and 5), 1 *L. crispatus* (LC2), 1 *L. mucosae* (LM2), 1 *L. vaginalis* (LV6) and *1 L. gasseri* (LG1)] before adding *G. vaginalis* ATCC 14018 and incubating the cultures for a further 20 hours. When comparing the concentrations of the four inflammatory cytokines assessed (IL-1α, IL-6, IL-8, IP-10) and anti-inflammatory IL-1 receptor antagonist (RA), it was found that 5/6 of the lactobacilli reduced inflammatory responses to *G. vaginalis* (Fig. 5). Using Student’s t test to compare log_10_-transformed cytokine concentrations, it was found that IL-6 concentrations were significantly lower following pre-incubation with LC2 (p = 0.0085, adjusted p = 0.0425), LV6 (p = 0.0091, adjusted p = 0.02275), LM2 (p = 0.0124; adjusted p = 0.0207), LG1 (p = 0.0179; adjusted p = 0.0224) and LJ2 (p = 0.0306; adjusted p = 0.0306), while pre-incubation with LJ5 significantly increased cytokine production relative to incubation with *G. vaginalis* alone (p = 0.0191; adjusted p = 0.0191). LC2 also significantly reduced IL-1α, IL-8 and IL-1RA production, while LJ2 reduced IL-8 and LG1 and LV6 reduced production of IL-1RA relative to *G. vaginalis* alone, although these changes were not significant after adjusting for multiple comparisons (p = 0.0337, p = 0.0196, p = 0.0312, p = 0.0124, p = 0.0217 and p = 0.0190, respectively).

### Inhibition of *G. vaginalis* growth

**Figure 6:**
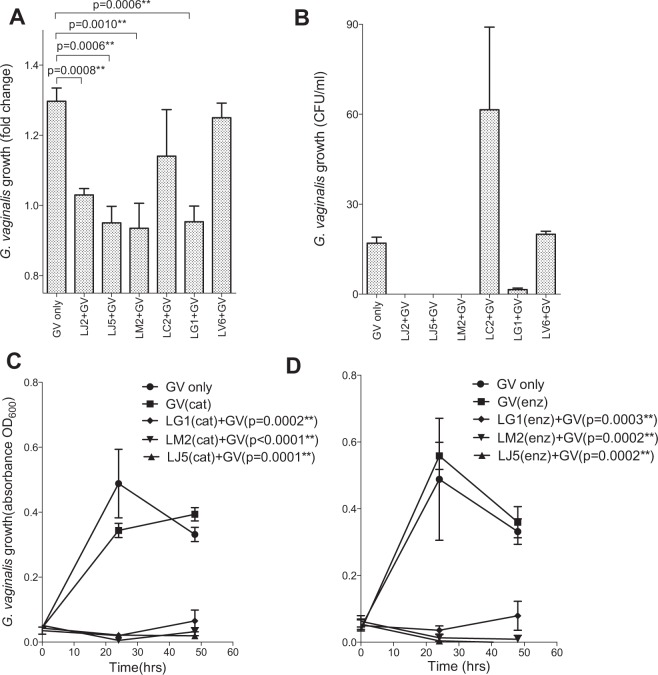
Inhibition of *Gardnerella vaginalis* growth by *Lactobacillus c*ulture supernatants. (**A**) Growth of *G. vaginalis* was determined by measuring the optical density of cultures at a wavelength of 600 nm (OD_600_), after culture with lactobacilli supernatants in duplicate within the same assay for 24 h at 37 °C. The fold change in growth of *G. vaginalis* only cultures was compared to those cultured with lactobacilli supernatants using an unpaired two-tailed t-test. (**B**) Colony forming units (CFU)/ml of *G. vaginalis* pretreated with lactobacilli supernatants on brain heart infusion (BHI) agar after incubation for 48 h at 37 °C were assessed in duplicate within the same assay. (**C**,**D**) L*actobacillus jensenii* (LJ)5, *L. mucosae* (LM)2 and *L. gasseri* (LG)1 were used to determine the mechanism underlying the inhibition of *G. vaginalis* by lactobacilli supernatant in triplicate within the same assay. (**C**) The effects of hydrogen peroxide reduction by catalase and (**D**) bacteriocin degradation by proteolytic enzymes in *Lactobacillus* culture supernatants on the growth of *G. vaginalis*. Lines indicate means and error bars indicate the standard deviations of technical replicates. Student’s t-tests were used for comparisons. **Adjusted p-values < 0.05 were considered statistically significant. CFU: colony forming units, LC: *L. crispatus*, LV: *L. vaginalis*, Cat: *catalase*, Enz = *proteolytic enzymes*.

The six *Lactobacillus* isolates above were selected for *G. vaginalis* inhibition assays, including 2 *L. jensenii* (LJ2 and 5), 1 *L. crispatus* (LC2), 1 *L. mucosae* (LM2), 1 *L. vaginalis* (LV6) and *1 L. gasseri* (LG1) strains. It was found that 4/6 isolates significantly inhibited *G. vaginalis* growth (Fig. 6A) and viability (Fig. 6B). The culture pH levels of the isolates that inhibited *G. vaginalis* growth and viability, LJ2, LJ5, LM2 and LG1, were significantly lower than those of the isolates that were not inhibitory, LC2 and LV6 [mean: 4.551 (range: 4.398–4.826) versus 5.478 (range: 5.283–5.673), respectively; p = 0.0082]. D-lactate, L-lactate and lactic acid levels did not differ significantly between the groups (p = 0.0703, p = 0.2711 and p = 0.0690, respectively), although the sample size was small and the differences in D-lactate and lactic acid concentrations approached significance. Furthermore, reduction of H_2_O_2_ and degradation of bacteriocins using proteolytic enzymes did not influence the inhibitory activities of LJ2, LM2 and LG1 (Fig. 6C,D), suggesting that culture medium acidification was the primary mechanism for *G. vaginalis* inhibition in these assays.

### Overall probiotic-relevant performance of isolates

A scoring system was devised to compare and rank the isolates based on all of the characteristics investigated. Each isolate was given scores out of a maximum score of three per characteristic, according to (1) growth rates; (2) culture pH acidification; the levels of (3) H_2_O_2_, (4) L- and (5) D-lactate produced, (6) ability to adhere to epithelial cells and (7) induction of inflammatory responses by CaSki cells (Table 2). Relative scores per category were assigned as follows: <25^th^ percentile (score = 0); 25^th^−50^th^ percentile (score = 1); 50^th^-75^th^ percentile score = 2); ≥75^th^ (score = 3). Lower pH levels and lower levels of inflammation were considered advantageous, so isolates were scored as follows: <25^th^ percentile (score = 3); 25^th^-50^th^ percentile (score = 2); 50^th^-75^th^ percentile score = 1); ≥75^th^ (score = 0). The *L. jensenii* strain (LJ5) ranked highest with a score of 18/21, higher than the probiotic isolates (*L. acidophilus* and *L. casei rhamnosus*). Interestingly, the top 10 isolates included 4 *L. jensenii*, 2 *L. crispatus*, 1 *L. mucosae*, and 1 *L. vaginalis* strain and the *L. acidophilus* probiotic isolates. The commercial vaginal probiotics were ranked 6^th^ (LA capsule; 13/21), 10^th^ (LA tablet; 12/21) and 20^th^ (LCR; 9/21). The performance of the LA strains was inconsistent between tablets and capsules, while both grew rapidly and lowered the culture pH levels effectively, both induced inflammatory responses and the capsule LA isolate was poorly adherent to epithelial cells, while the tablet LA isolate produced very little H_2_O_2_ and L-lactate. The LCR probiotic strain fared worse with poor adhesion to epithelial cells and low H_2_O_2_, L- and D-lactate production in comparison to clinical isolates. Similar rankings were obtained if H_2_O_2_ was omitted, with the top 10 containing 3 *L. jensenii*, 2 *L. crispatus*, 1 *L. mucosae*, 2 *L. vaginalis*, and the 2 *L. acidophilus* probiotic isolates.

**Table 2:** Overall characteristics of clinical *Lactobacillus* isolates, commercial probiotics and reference strains.

Isolate	Growth rate	Culture pH	Adhesion	Hydrogen peroxide	L-lactate	D-lactate	Inflammatory response	Sum
LJ 5	3	2	3	2	3	2	3	18
LM 2	2	1	3	3	2	1	3	15
LJ 4	1	3	2	3	3	0	3	15
LJ 1	3	3	3	3	0	2	0	14
LC 7	3	3	1	3	0	2	1	13
LA capsule	2	3	1	2	2	2	1	13
LC 6	3	3	2	1	2	0	2	13
LV 3	3	0	1	0	2	3	3	12
LJ 6*	0	2	0	3	3	3	1	12
LA tablet	3	2	3	1	0	3	0	12
LV 2	2	0	2	0	2	3	2	11
LG 2*	0	2	2	2	3	2	0	11
LJ 3	0	2	0	2	3	3	1	11
LJ 2	1	0	3	2	3	1	0	10
LM 4	2	1	2	3	0	2	0	10
LV 5	1	0	1	1	3	3	0	9
LM 1	1	1	2	2	0	2	1	9
LC 4	1	2	3	0	0	1	2	9
LC 1	3	3	3	0	0	0	0	9
LCR	3	3	1	0	0	1	1	9
LM 3	1	1	1	3	0	0	2	8
LC 5	0	1	2	2	0	0	3	8
LC 8*	2	2	0	1	0	1	2	8
LC 3	0	2	2	0	2	1	1	8
LV 4	1	0	1	0	0	3	2	7
LV 1	1	1	0	0	2	1	2	7
LC 2	2	0	0	0	0	2	2	6
LV 6	0	0	1	1	0	0	3	5
LG 1	0	1	0	2	0	0	2	5
LC 9*	2	1	0	0	0	1	1	5

LA: *Lactobacillus acidophilus*; LC: *Lactobacillus crispatus*; LCR: *Lactobacillus casei rhamnosus*; LG: *Lactobacillus gasseri*; LM: *Lactobacillus mucosae*; LV: *Lactobacillus vaginalis*; LJ: *Lactobacillus jensenii*. *ATCC reference strains.

## Discussion

BV, an imbalance in vaginal microbiota that predisposes women to HIV infection and reproductive complications, remains prevalent, in part because standard-of-care antibiotic therapy is largely ineffective^14,32^. Adjunctive probiotic treatment for BV may promote vaginal recolonization with healthy lactobacilli, however BV treatment outcomes have been heterogeneous in probiotic clinical trials. Here, we show that the performance of the only vaginal probiotics available on the South African market were highly varied based on the criteria evaluated in this study, providing a possible explanation for the heterogeneous efficacy of probiotic treatment in BV. Neither *L. acidophilus* nor *L. casei rhamnosus* species predominate in the FGTs of women with healthy microbiota^27^ and were not isolated from the cohort of women included in this study, suggesting that these species might not be ideal for treatment of BV in South African women. In this study, we evaluated *Lactobacillus* species that have been associated with vaginal health, including *L. crispatus*, *L. gasseri, L. jensenii, L. mucosae* and *L. vaginalis*. *L. iners* was not included as this species is associated with increased risk of conversion from a healthy to a dysbiotic vaginal microbiome^33^, and acquisition of STIs^34^. We found that several of the clinical isolates performed better than the probiotic strains, demonstrating the potential to improve existing probiotic formulations and treatment outcomes using novel clinical isolates. We also found that, although some *Lactobacillus* isolates induced inflammatory responses when cultured with ectocervical epithelial cells in isolation, when we pre-incubated the cells with a subset of six isolates prior to addition of *G. vaginalis*, 5/6 of the lactobacilli significantly reduced pro-inflammatory cytokine responses to *G. vaginalis*.

Several antimicrobial and other characteristics that are thought to be important for effective probiotic activity were evaluated for each *Lactobacillus* isolate^16,18,20^. Adhesion, thought to be mediated by specialized pili^35^, is an essential probiotic property as it enables the bacteria to persist in the FGT^36^. In this study, there were no significant differences in the level of adhesion between species. Larger bacterial size has been linked to superior adhesion capabilities^30^, however the relative length of bacteria in this study did not correlate significantly with adhesion. Moreover, although it has been reported that rapid bacterial multiplication leads to a high adhesion capacity^30^, in the present study, bacterial growth rates were not significantly associated with adhesion. H_2_O_2_ production differed between species, with *L. mucosae* isolates producing the most H_2_O_2_, followed by *L. jensenii* isolates, while the others produced minimal H_2_O_2_. The importance of H_2_O_2_ in protection against pathogens in the FGT is controversial. Earlier clinical studies found that women with H_2_O_2_-producing lactobacilli were at reduced risk of dysbiosis^8,18,37^. However, it has since been found that, under hypoxic conditions like those found in the vagina, lactobacilli produce very little H_2_O_2_^38^ and H_2_O_2_ may not be microbicidal at physiological concentrations^19,39^. Furthermore, at microbicidal concentrations H_2_O_2_ inactivated lactobacilli more effectively than BV-associated bacteria *in vitro*^19,39^. It is important to note that, using an *in vitro* model to evaluate the characteristics of *Lactobacillus* isolates has limitations as this environment does not perfectly mimic *in vivo* conditions.

In contrast, lactic acid at physiological concentrations was shown to be microbicidal against pathogenic BV-associated bacteria (including *G. vaginalis*, *P. bivia* and *P. corporis*), but not vaginal lactobacilli^16,17,19,39^. Physiological concentrations of lactic acid have also been shown to have broad-spectrum virucidal activity against HIV that is dramatically more rapid and potent than media acidified to the same pH with HCl or acetic acid^15,40^. Interestingly, all isolates in this study produced D-lactate with no significant differences between species. However, L-lactate production was highly varied, with *L. jensenii* isolates producing the most L-lactate in both MRS under anaerobic conditions and in DMEM under aerobic conditions overall. The relative importance of L-lactic acid versus D-lactic acid is also controversial. L-lactic acid was found to be 17-fold more potent than D-lactic acid in inactivating HIV_BaL_ *in vitro*^15,40^. However, D-lactic acid was found to have a greater inhibitory effect on *C. trachomatis* infectivity than L-lactic acid, which was partly^41^ or entirely^39^ pH-dependant.

Similar to the finding that lactic acid concentrations correlate inversely with pH in the FGTs of women with *Lactobacillus*-dominated microbiota^31^, we found that lactic acid concentrations correlated significantly with culture pH. As found in the present study, a previous study showed that the vaginal acidity achieved by *L. crispatus* was the highest compared to *L. jensenii* and *L. gasseri*^27^. Interestingly, in the present study, the *L. casei rhamnosus* probiotic isolate was associated with the lowest culture pH, followed by *L. acidophilus* probiotic.

We measured inflammatory responses to lactobacilli, as genital inflammation has been found to increase risk of HIV acquisition in women^21^ and it is thus essential that probiotics induce little or no inflammation. Most of the isolates in isolation induced low levels of cytokine production by CaSki cells following a 24 h incubation period. However we also found that most of the lactobacilli evaluated were able to significantly suppress inflammatory cytokine production by CaSki cells in response to the BV-associated pathogen, *G. vaginalis*. *In vivo*, in the same cohort from whom the lactobacilli were isolated, we found that endogenous *L. reuteri, L. gasseri, L. crispatus* and *L. jensenii* were all significantly associated with low inflammation in the FGT^42^. Other studies have reported that lactobacilli and lactic acid suppress inflammatory responses to pathogens and pattern recognition receptor ligands *in vitro*^22,26^. It is interesting that, in this study, some of the lactobacilli in isolation were able to induce increases in pro-inflammatory cytokine production and 1/6 of the lactobacilli included in the *G. vaginalis* co-culture experiment had an additive effect on cytokine induction. Additionally, anti-inflammatory IL-10 production was not detected in these cultures and IL-1RA production was suppressed by lactobacilli. Another study showed that, while *L. rhamnosus* and *L. reuteri* suppressed the expression of certain inflammatory mediators by vaginal epithelial cells in response to *Candida albicans* [including nuclear factor-kappa B inhibitor kinase alpha, toll-like receptor (TLR)-2, TLR-6, IL-8, and TNF-α], the lactobacilli also induced expression of pro-inflammatory cytokines IL-1α and IL-1β^43^. The authors suggested that the lactobacilli may suppress inflammatory responses induced by the NF-κB signal transduction pathway, but induce other inflammatory responses via an alternate signal pathway, such as the mitogen activated protein kinase and activator protein-1 (MAPK/AP-1) signal transduction pathway^43^. Similarly Rose *et al*. (2012) found that lactobacilli induced non-significant increases in the production of some inflammatory cytokines by vaginal epithelial cells cultured in transwell culture systems^44^. Proteins present in the peptidoglycan layer of the *Lactobacillus* cell wall and lipoteichoic acids present in the cell membrane may have inflammatory properties and the inflammatory nature of particular *Lactobacillus* strains has been found to be partly dependent on the structure of the peptidoglycan and the presence of exopolysaccharides which prevent the interaction of these TLR agonists with pattern recognition receptors^45^. Therefore it is not surprising that some isolates are capable of inducing inflammatory responses. Lactic acid itself was found to have direct pro-inflammatory effects on immune and vaginal epithelial cells in some studies^46,47^, but anti-inflammatory effects in others^22^. In this study, we found that neither D-lactate nor L-lactate production correlated with cytokine production by CaSki cells in response to the lactobacilli isolates. In addition to influencing inflammatory pathways directly, lactobacilli may alter inflammatory responses to pathogens by means of competitive exclusion, preventing pathogens from interacting with vaginal epithelial cells^20,30^. Interestingly, although the lactic acid concentrations measured in these cultures were lower than those present in FGT secretions^31^, we still observed suppression of pro-inflammatory responses to *G. vaginalis* and inhibition of *G. vaginalis* growth by *Lactobacillus* isolates and culture supernatants, respectively. Inflammatory cytokine induction by some of the lactobacilli demonstrates the need to evaluate inflammatory profiles of bacterial isolates being considered for vaginal probiotic therapy. A limitation of this study is that an ectocervical cancer cell line was used to evaluate lactobacilli adhesion and inflammatory properties to allow for high-throughput screening. However, the inflammatory properties of these cells may differ from healthy cells and it will thus be important to confirm that probiotic candidates do not induce inflammatory responses using other models.

Interestingly, the characteristics of the *Lactobacillus* isolates from South African women investigated in this study differed in some respects to the characteristics of isolates from other geographical regions and women of different ethnicities^27,28^. While studies in other regions, including America and Spain^37,48^, have found that *L. crispatus* strains produce high levels of H_2_O_2_ and lactic acid, this study found that these South African *L. crispatus* isolates generally produced relatively little of these antimicrobials. Similarly, major geographical and ethnic differences in the vaginal microbiome have been noted. African women were found to have a low abundance of *Lactobacillus* that was not associated with sexual behavior, contraceptive usage, or demographic characteristics^4^.

A lower frequency of *L. crispatus* and *L. jensenii* has been observed in black and Hispanic women compared to white and Asian women in the United States, suggesting that host genetic or dietary factors may play a role in bacterial colonization of the FGT^5,27,49^. However it is also possible that the characteristics of the predominant *Lactobacillus* strains present in certain populations influence the species distribution.

According to the characteristics that were assessed in this study that may influence *Lactobacillus* antimicrobial activity (including growth rates, culture pH, adhesion, H_2_O_2_, lactic acid and inflammatory responses), several South African clinical isolates performed better than the commercial probiotic *Lactobacillus* isolates evaluated. Of the top ten *Lactobacillus* isolates, *L. jensenii* and *L. crispatus* were well represented, while *L. mucosae* and *L. vaginalis* strains were mostly absent. Five clinical isolates performed better than the probiotic *L. acidophilus s*train, suggesting that there is indeed potential to improve existing probiotics that may, in turn, improve BV treatment outcomes.

## Methods

### Study participants

A cohort of 149 young women (16–22 years old) was recruited as part of the Women’s Initiative in Sexual Health (WISH) study in Cape Town^29^. Demographic data was collected and lateral wall and vulvovaginal swabs were obtained for testing of STIs (*C. trachomatis*, *N. gonorrhoeae, T. vaginalis, M. genitalium*, HSV-1, HSV-2 and *T. pallidum*) by PCR, and candidiasis and BV by Gram stain. This study was approved by the University of Cape Town Human Research Ethics Committee and all methods were performed in accordance with the relevant guidelines and regulations. Women older than 18 years old provided written informed consent, while those 16–17 years provided assent and written informed consent was obtained from their parents or legal guardians.

### Bacterial isolation and culture

Cervicovaginal fluid was collected in menstrual cups (Softcup®, Evofem Inc, USA), diluted in phosphate buffered saline (PBS) and frozen in 20% glycerol at −80 °C. Cervicovaginal fluid was thawed and cultured in sterile de Man, Rogosa, Sharpe (MRS) broth anaerobically for 48 h at 37 °C, streaked on MRS-agar and incubated anaerobically at 37 °C for 48 h. Morphologically distinct single colonies were picked and inoculated in MRS broth and incubated anaerobically at 37 °C for 72 h. Bacterial isolates (n = 50) were cultured from 20 participants and identified using MALDI biotyping (Bruker Daltonik, USA). From these, 23 isolates from 9 women were identified as *Lactobacillus* species and were included. Microscopy was used to measure the length of 5 bacteria per isolate (Leica ICC50 HD, Leica Microsystems, Germany). Growth rates were evaluated by inoculating MRS broth with 4.18 × 10^6^ CFU^47^ of each isolate in triplicate and measuring the OD_600_ of cultures over time. The area under the curve (AUC) was calculated for each isolate between 0 and 12 h, after which stationary phase was reached.

### Bacterial adhesion to genital epithelial cells

The ability of the lactobacilli to adhere to human ectocervical epithelial cells, CaSki (CRL-1550, ATCC, USA), was evaluated as described previously^50^. CaSki cells were grown to 80% confluency in complete cell culture medium [Dulbecco’s Modified Eagle’s Medium (DMEM) with L-Glutamine (Lonza, Switzerland), 10% fetal calf serum (Capricorn-Scientific, Germany) and 1% penicillin (50U/ml) and streptomycin (50U/ml; Sigma-Aldrich, USA)] in 24 well plates and then washed with PBS. Bacteria adjusted to OD_600_ of 0.1 ± 0.01 in antibiotic-free cell culture medium were added and incubated for 2 h at 37 °CC, 5% CO_2_. Culture medium was removed and plates were washed 3 times with PBS to remove unbound bacteria. 0.1% TritonX-100 (Sigma-Aldrich, USA) was added and the monolayers were lifted with cell scrapers. Serial dilutions were plated on MRS agar and colonies were counted following anaerobic incubation at 37 °C for 48 h. CFU/ml was calculated and expressed as a percentage of the baseline CFU/ml added to each monolayer. This experiment was repeated three times.

For visual confirmation of bacterial adhesion to CaSki cells, bacteria were added to cell monolayers in Nunc^TM^ Lab-Tek^TM^ II Chamber Slides (Thermo Fisher Scientific Inc., USA) and incubated for 2 h at 37 °C, 5% CO_2_. Cell culture medium was removed and wells were washed 3 times with PBS. Chambers were removed before each slide was heat-fixed and Gram-stained. Representative images were captured (Leica ICC50 HD, Leica Microsystems, Germany).

### Hydrogen peroxide production

H_2_O_2_ production was evaluated in aerobic cultures in order to assess the maximal H_2_O_2_ production capacity of the isolates. Cultures were incubated aerobically with agitation at 170 rpm for 3 h at 37 °C. Aliquots were collected hourly for 3 h, centrifuged and the supernatants stored at −80 °C. pH levels of the cultures were measured using a pH meter (Jenway Bench pH Meter 2510, Bibby Scientific, UK). Measurement of *Lactobacillus* H_2_O_2_ concentrations was performed using a tetramethylbenzidine assay in duplicate as described previously^51^.

### Inflammatory cytokine responses to lactobacilli

CaSki monolayers were grown to near confluence in 24 well plates. Culture medium was removed and 4.18 × 10^6^ CFU of each bacterial species or OD_600_ of 0.1 ± 0.01 adjusted bacteria were added to the cells in antibiotic-free cell culture medium (DMEM with L-Glutamine, 10% fetal calf serum) and incubated aerobically at 37 °C for 24 h under 5% CO_2_. Culture supernatants were removed, centrifuged at 6000xg and stored at −80 °C. CaSki cell viability in these co-cultures was confirmed using an MTT assay (Roche Diagnostics, Germany). In order to account for the impact of cleavage of the yellow tetrazolium salt MTT to purple formazan crystals by metabolically active lactobacilli, lactobacilli-only cultures incubated in antibiotic-free cell culture medium under aerobic conditions were included as controls. In all cases, the metabolic activity of the CaSki cells in co-culture with the lactobacilli was either comparable to or higher than the CaSki cells-only controls. A Magnetic Luminex Screening Assay kit (R&D Systems, Minneapolis, USA) was used to measure the concentrations of 13 cytokines (TNF-α, IL-1α, IL-1β, IL-6, IL-17A, IFN-γ, RANTES, IL-8, IP-10, MIP-3α, MIP-1α and MIP-1β, IL-10). To evaluate whether cytokine responses to *G. vaginalis* were modulated by *Lactobacillus* isolates, CaSki cells were pre-incubated with 4.18 × 10^6^ CFU of six *Lactobacillus* isolates [2 *L. jensenii* (LJ2 and 5), 1 *L. crispatus* (LC2), 1 *L. mucosae* (LM2), 1 *L. vaginalis* (LV6) and *1 L. gasseri* (LG1) strains] for 5 hours under the same conditions as described above. *G. vaginalis* ATCC 14018 (4.18 × 10^7^ CFU) was then added to each well and cultures were incubated for a further 20 hours. Supernatants were collected and processed as above and a Magnetic Luminex Screening Assay kit (R&D Systems, Minneapolis, USA) was then used to measure IL-1α, IL-6, IL-8, IP-10 and IL-1RA concentrations. Data was collected using a Bio-Plex^TM^ Suspension Array Reader and a 5 parameter logistic regression was used to calculate cytokine concentrations from the standard curves using BIO-plex manager software (version 4; Bio-Rad Laboratories Inc®, USA). Cytokine concentrations below the detectable limit were assigned the value of half the lowest recorded concentration of that cytokine.

### D- and L-lactic acid production

Following a 24 h incubation period at 37 °C under anaerobic conditions, 4.18 × 10^6^ CFU of each isolate was added to 15 ml MRS broth and incubated for an additional 24 h at 37 °C under anaerobic conditions. Supernatants were collected and D-and L-lactate concentrations were measured in duplicate using D-Lactate Colorimetric and Lactate Assay kits according to the manufacturer’s instructions (Sigma-Aldrich, USA). Both D- and L-lactate concentrations were also measured in lactobacilli-CaSki cell co-cultures following aerobic incubation at 37 °C for 24 h under 5% CO_2_ using D-Lactate Colorimetric and Lactate Assay kits according to the manufacturer’s instructions (Sigma-Aldrich, USA)_._

### *G. vaginalis* inhibition assay

Six *Lactobacillus* isolates [2 *L. jensenii* (LJ2 and 5), 1 *L. mucosae* (LM2), 1 *L. crispatus* (LC2), 1 *L. gasseri* (LG1), 1 *L. vaginalis*, (LV6)] were cultured in MRS for 24 h under anaerobic conditions. Cultures were standardized to 4.18 × 10^6^ CFU/ml in MRS and incubated for an additional 20 h at 37 °C under anaerobic conditions, after which they were filtered using 0.2 μm cellulose acetate filters (Sigma-Aldrich, USA). Following 48 h incubation in brain heart infusion (BHI) broth at 37 °C under anaerobic conditions, *G. vaginalis* was standardized to 1 × 10^8^ CFU/ml and cultured with each lactobacilli cell-free supernatant anaerobically for 24 h at 37 °C. Absorbance at OD_600_ was measured at baseline and 24 h after incubation. Following the 24 h incubation, *G. vaginalis* cultures were plated on BHI agar and incubated for 24 h at 37 °C under anaerobic conditions, after which CFUs were counted^12^.

### Effect of hydrogen peroxide and bacteriocins on *G. vaginalis* inhibition

Three *Lactobacillus* isolates (LJ2, LM2 and LG1), that were found to inhibit *G. vaginalis* growth, were standardized to 4.18 × 10^6^ CFU/ml and cultured in MRS broth for 20 h at 37 °C under anaerobic conditions. To abrogate the possible effect of H_2_O_2_ and bacteriocins, lactobacilli cell-free supernatants and MRS were pre-treated for 1 h at 37 °C with catalase (100 µg/ml) or proteolytic enzymes [trypsin (200 µg/ml), pepsin (200 µg/ml), pronase (200 µg/ml) and proteinase K (100 µg/ml)]. Pre-treated lactobacilli cell-free supernatants, pre-treated MRS and untreated MRS were then added 1 × 10^8^ CFU/ml of *G. vaginalis*. Absorbance at OD_600_ was measured at baseline, 24 and 48 h after incubation at 37 °C under anaerobic conditions^12^.

### Statistical Analysis

GraphPad Prism 5® (GraphPad Software, USA), STATA version 11.0 (StataCorp, USA), and R were used for statistical analyses. Mann Whitney U and Student’s t-tests were used for unpaired comparisons of non-parametric and parametric data, respectively; Spearman Rank test was used for non-parametric correlations. A false-discovery rate (FDR) step-down procedure was used to adjust p-values for multiple comparisons and adjusted p-values < 0.05 were considered statistically significant. Unsupervised hierarchical clustering was used to evaluate the relationship between lactobacilli and cytokine profiles. To compare overall inflammatory cytokine responses, confirmatory factor analysis was used to generate inflammatory factor scores, which are linear combinations of the concentrations of each inflammatory cytokine (IL-1α, IL-1β, IL-6, IL-8, IP-10, MIP-1α, MIP-1β, MIP-3α, RANTES) in the factor, weighted according to their factor loadings.

### Ethics approval and consent to participate

The parent study was approved by the University of Cape Town (UCT) human research ethics committee (UCT HREC: 267/2013). The microbiological sub-study was approved by the UCT human research ethics committee (UCT HREC: 551/2016). Women older than 18 years provided written informed consent, while those who were 16–17 years old provided assent and written informed consent was obtained from their parents or legal guardians.

## Data Availability

The datasets used and/or analysed during this study are available from the corresponding author on reasonable request.
